# Intrafamily Transmission of HCV Need to More Discussion

**Published:** 2011-01-01

**Authors:** S M Alavian

**Affiliations:** 1 Baqiyatallah Research Center for Gastroenterology and Liver Diseases, Baqiyatollah University of Medical Sciences, Tehran, Iran

**Keywords:** Intrafamily, Transmission, HCV, Iran

Dear Editor,

I read with interest the recently published article by Imani et al.,[1] in your journal, in which five out of the 230 (2.17%) tested household contacts were seropositive. We do not have enough data regarding family prevalence of hepatitis C virus (HCV) infection in the literature. Blood and blood products transfusion used to be the main risk factor for transmission of HCV, yet the main risk factor has recently changed to intravenous drug abusing (Injecting drug users; IDUs).[2][3][4] The prevalence of HCV infection in general population of I.R. Iran is low[5] and it means that the main risk factor for transmission of HCV infection, IDUs, is low in our general population. The study reveals that we should be aware of transmission of HCV infection in families, but I would like to add some points for more discussion about this issue. The risk of transmission of HCV by sexual rout is very low;[6] the other risk factors such as sharing the razors and tooth brushes may be responsible as well. I would like to ask the physicians to train the family of patients more regarding prevention of HCV transmission. Sexual health precautions and avoiding sexual contact during the menstrual period, according to Islam rules, is mandatory. Another point about the study by Imani et al. in which four out of the mentioned five seropositive household contacts (80%) were the cases' wives and the remaining one (20%) was one of their sister, is the probability of IDUs history in the family positive for IDU especially in the one infected sister which means the transmission had occurred due to a high risk behavior and not intrafamily.[6][7] We do not have any information about this risk factor in positive family cases in the article.
